# Motion cues that make an impression^☆^: Predicting perceived personality by minimal motion information

**DOI:** 10.1016/j.jesp.2013.08.002

**Published:** 2013-11

**Authors:** Markus Koppensteiner

**Affiliations:** Department of Anthropology/Human Behavior Research, University of Vienna, Althanstrasse 14, 1090, Vienna, Austria

**Keywords:** Body motion, Motion analysis, Nonverbal communication, Social cognition, Impression formation, Politics

## Abstract

The current study presents a methodology to analyze first impressions on the basis of minimal motion information. In order to test the applicability of the approach brief silent video clips of 40 speakers were presented to independent observers (i.e., did not know speakers) who rated them on measures of the Big Five personality traits. The body movements of the speakers were then captured by placing landmarks on the speakers' forehead, one shoulder and the hands. Analysis revealed that observers ascribe extraversion to variations in the speakers' overall activity, emotional stability to the movements' relative velocity, and variation in motion direction to openness. Although ratings of openness and conscientiousness were related to biographical data of the speakers (i.e., measures of career progress), measures of body motion failed to provide similar results. In conclusion, analysis of motion behavior might be done on the basis of a small set of landmarks that seem to capture important parts of relevant nonverbal information.

## Highlights

• Body movements of politicians making a speech were captured.
• Short video clips of these speeches were rated on personality.
• Motion cues were related to personality traits and measures of career progress.
• Simple motion cues and simple cognitive processes may guide impression formation.
• Simple motion cues may play an important role in human communication.

## Introduction

Brief displays of behavior (i.e., “thin slices”) can provide a sufficient source of information to assess other people's personalities (e.g., Ambady, Bernieri, & Richeson, 2000; Borkenau, Mauer, Riemann, Spinath, & Angleitner, 2004; Gifford, 1994; Kenny, Horner, Kashy, & Chu, 1992). Moreover, nonverbal and appearance cues displayed during such thin slice evaluations do not only have an impact on how people judge their interaction partners; they even have more far reaching consequences. In the public arena, they can affect politicians' perceived competence and personality, help to build social bonds to an audience, and guide people's voting decisions (Antonakis & Dalgas, 2009; Ballew & Todorov, 2007; Poutvaara, Jordahl, & Berggren, 2009; Stewart, Waller, & Schubert, 2009). It is of great interest, therefore, to elucidate by which cues first impressions are formed.

While facial expressions and certain physiognomic features play an important part in nonverbal communication, research suggests that people are attentive to motion cues. Johansson (1973), for instance, demonstrated that a set of moving dots obtained by attaching reflective markers to the major joints of a person is still recognized as a human body in motion. Building on Johansson's approach other researchers even found that such “point light” displays convey socially relevant information (e.g., Chouchourelou, Matsuka, & Shiffrar, 2006; Clarke, Bradshaw, Field, Hampson, & Rose, 2005; Pollick, Patterson, Bruderlin, & Sanford, 2001; Thoresen, Vuong, & Atkinson, 2012).

This is also supported by a diversity of alternative methods that have been devised to investigate human body motion. Variations in frequency and duration of motion and other kinematic features play a role in mating behavior (Bente, Donaghy, & Suwelack, 1998; Grammer, Honda, Juette, & Schmitt, 1999), affect judgments of attractiveness (Neave et al., 2011), are linked to self-rated and observer-rated personality (i.e., sensation seeking and Big Five) of people performing dances (Hugill, Fink, Neave, Besson, & Bunse, 2011; Luck, Saarikallio, Burger, Thompson, & Toiviainen, 2010) and elicit different attributions of personality and health to politicians making a speech (Koppensteiner & Grammer, 2010, 2011; Kramer, Arend, & Ward, 2010).

The current study builds on this work and presents a parsimonious way of capturing simple patterns of motion. The methods I introduce are designed for unobtrusive behavior analyses via video data and may be used to examine behavior that is displayed under “natural conditions.” As a first step to test the presented methods' applicability body movements of politicians (i.e., silent short video clips of public speeches with speakers that were unknown to the participants) were captured and related to ratings of personality and some biographical data such as the number of leadership roles the politicians had during their careers. The process of behavior encoding was done by placing a small set of only four landmarks onto some “hot spots” of the speakers' bodies. Position shifts of these spots, which corresponded to the body motion occurring, were in part recorded by software routines based on optical flow (see Methods section), yet corrections with the computer mouse were also necessary, since the detection algorithm is error-prone.

The study pursues several aims. It intends to show that under some conditions body motion can be analyzed without creating abstract representations (i.e., point light animations, stick figures etc.) of a stimuli's behavior, that encoding can be simplified by using a minimal set of landmarks, that such a small set of landmarks captures enough relevant information to predict first impressions and that such first impressions are also revealing about how people behave in other contexts (i.e., public behavior in this study). Taking into account that very brief displays of behavior elicit attributions of personality, I hypothesize that the cues people use for their judgments are simple and appear consistently (i.e., certain patterns are displayed repeatedly). For this reason the analysis tools are designed to extract motion cues, which give a kind of global description of a speaker's behavior. Further, I speculate that people's impressions are guided by simple cognitive processes, which make them associate particular motion patterns to particular personality traits. More details on the last point are given in the discussion.

## Methods

### Participants

Eighty-one participants (43 females with a mean age of *M* = 23.1, *SD* = 4.3; 38 males with a mean age of *M* = 23.9, *SD* = 3.7) were recruited in locations throughout the University of Vienna. I approached participants personally and asked if they wanted to take part in a rating experiment. Ratings were done in room equipped with several computers. Participants were not reimbursed.

### Stimulus preparation and procedure

I selected 40 speeches from the German Houses of Parliament (20 female and 20 male speakers), and randomly extracted brief video segments from each of the speeches (see also Koppensteiner & Grammer, 2010, 2011). This resulted in 40 short video clips with a length of 16 s. The lower portion of the video clips was cut off to remove captions giving information about a speaker's party. The speakers were ordinary members of the German Bundestag, which were unknown to Austrians. In addition, the participants of the rating-experiment had been asked to name any of the persons they had judged and in all cases they were unable to do that.

To encode the body movements of the speakers I developed the Speech Analyzer program, which is able to capture motion from video files. The software runs through a movie stepwise (here: steps of three frames) and uses motion tracking computer algorithms^1^ (i.e. optical flow) to trace the trajectory of an object. Where the algorithm loses track of an object, the position of the corresponding landmark (i.e. dot placed onto the object by the program) has to be corrected by hand using the computer mouse. In short, motion occurring between single pictures of the movies examined here was stored as a sequence of two dimensional landmark coordinates. For this study four landmarks were used: one placed on the forehead, one for the right shoulder (provides an estimate of torso rotations), one for the right and one for the left hand.

Stimuli were presented with a rating program. On the left side of the program's user interface the movies were displayed. The 20 bipolar items displayed on the right side were based on a brief German version of the NEO-FFI personality inventory (Borkenau & Ostendorf, 1991). The underlying psychological constructs of this questionnaire are extraversion, agreeableness, conscientiousness, neuroticism or emotional stability, and openness. For its computerized version I inserted slider controls between the pairs of adjectives, which enabled the participants to complete their ratings by dragging a bar to the right or to the left (i.e., to the item which appeared most appropriate to them) with a computer mouse. The original paper and pencil version of the questionnaire is based on a seven-point Likert scale. To create more variance for the ratings the slider control was divided into 100 subunits ranging from 0 (i.e., maximum rating of item on the left side of the slider control) to 100 (i.e., maximum rating of item on the right side of the slider control) with 50 being the neutral position. Each participant rated a subset of eight speakers, which was randomly selected from the 40 movies.

### Biographical data

In order to extend the empirical basis of the study I did not only collect ratings of personality, but also included information about the speakers' careers. To this end I used the online biographies of the politicians, which are presented at the homepage of the German Houses of Parliament (i.e., German Bundestag). With this data I created three measures, which in part may reflect the politicians' status, sense of self-importance, and aspiration to power.

As a simple estimate of career progress I subtracted the year, when the politicians became a member of the Bundestag from the year they became a member of a party (i.e., can be considered as starting point of their career in politics). I also determined the number of associations, clubs, committees and supervisory boards the politicians have been and still are members and divided it by the politicians' age. In order to provide an estimate of the politicians' claim to leadership I counted all leadership positions and deputy leadership positions and divided it by the politicians' age.

### Motion analysis

**Fig. 1:**
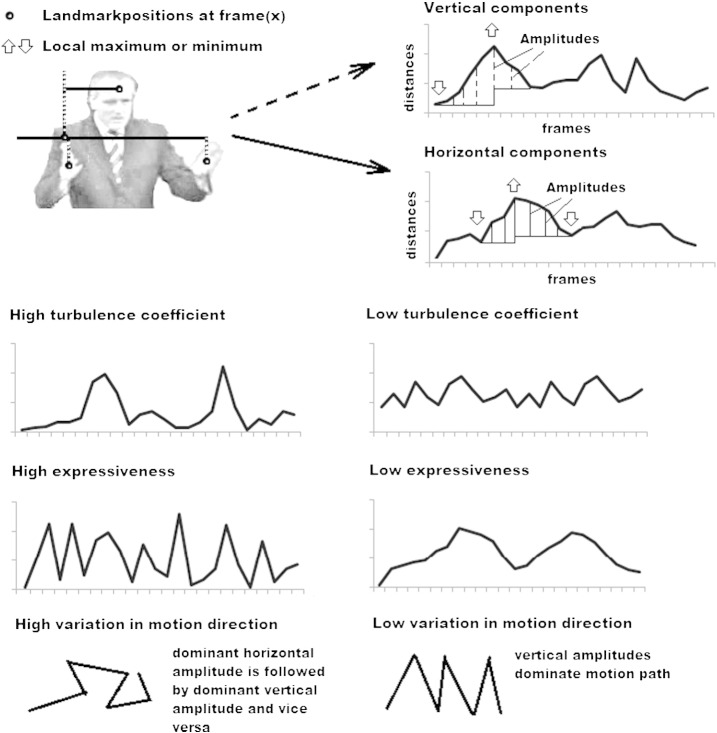
Simple illustrations of behavior encoding and motion analysis.

The speaker's behavior was turned into four time series (i.e., according to the four landmarks) of two dimensional coordinates. Coordinates of the right shoulder served as reference point from which the *x*-coordinate and the *y*-coordinate of the other landmarks were subtracted. Motion of the right shoulder itself was derived by subtracting its coordinates from the coordinate origin (upper left corner). In conclusion, coordinate data representing the position shifts of the body was translated into successions of horizontal and vertical amplitudes (see Fig. 1). From this data I extracted “motion cues”, which are presented below.

#### Sum of amplitudes

Motion behavior was turned into a succession of amplitudes. Summing these amplitudes (i.e., decomposed into horizontal and vertical components) provided an estimate of a speaker's overall activity. Amplitudes were corrected for body height (i.e., largest distance between forehead landmark and lectern).

#### Turbulence coefficient

The coefficient of variation or turbulence coefficient (i.e., ratio of standard deviation to the mean) is a normalized measure of dispersion that estimates whether a speakers' behavior follows a more or less regular pattern or whether there are irregular phases of low and high activity.

#### Expressiveness (or relative overall velocity)

This method estimates a speaker's tendency to produce movements of high or low velocity (see Koppensteiner & Grammer, 2010) relative to overall activity (i.e. the influence of a speaker's overall amplitude was removed from this measure). A tendency to produce smooth movements yields a low value for expressiveness, while a tendency to produce movements that may be characterized as “twitching” or “jerky” yields a high value for expressiveness.

#### Variation in motion direction

This measure counts how often the maximum amplitude switches from a horizontal to a vertical component and vice versa, thereby giving a rough estimate of the amount of changes in motion direction.

## Results and discussion

Each video clip was rated on 20 items by a range from 14 to 19 participants (*M* = 16.2). The ratings were averaged (i.e., using the median) with corresponding items (i.e., those building a personality dimension) summed. Thus, the ratings of every single speaker were reduced to five values, each representing one of the Big Five personality dimensions.

Intercorrelations between the Big-Five personality dimensions found a strong relationship of openness with conscientiousness, agreeableness, and emotional stability, and an equally pronounced relationship between emotional stability and agreeableness. A moderate relationship was also found between extraversion and emotional stability (Table 1). This might interpreted as a weakness of the questionnaire, because the Big Five dimensions should constitute five independent personality factors. However, it is seems very plausible and is a common finding also that nonverbal behavior cannot be divided into chunks of independent categories. Smiles, for instance, are often related to more than one personality trait (e.g., Gifford, 1994; Naumann, Vazire, Rentfrow, & Gosling, 2009). For such a reason — just to give an example — in the current study ratings of emotional stability might be strongly related to agreeableness, because a friendly politician might also appear emotionally stable.

Analysis of interdependencies between “measures of career progress” revealed relatively small correlations of “entry into parliament” (i.e., years needed to become member of the Bundestag) with the number of membership positions (*r_s_* = − .15, *p* = .34) and the number of leadership positions of a politician (*r_s_* = − .16, *p* = .32). A distinct relationship, however, was found between membership and leadership positions (*r_s_* = .42, *p* = .00). This indicates that politicians who are members in a great number of committees, clubs and associations also often hold leadership positions in such organizations.

Correlations of personality ratings with the politicians' autobiographical data provided noteworthy results. Speakers that received high values for the personality dimensions of openness and conscientiousness seemed to need more years to become a member of the Bundestag and hold and held less membership positions than speakers that received low ratings for openness and conscientiousness (Table 1). Although these results need additional empirical support, they indicate that ratings of high openness and high conscientiousness are more than simple attributions. To a certain degree they can be used to determine who is “slow” or “fast” with regard to career progress the way it is defined here. In particular for conscientiousness this appears to make sense, because a large number of public positions are in conflict with the amount of attention that can be paid to each one of these. Also, the findings are a hint that appearance and behavioral cues reveal something about the actual personality of politicians.

Correlations between measures of body motion found a wide range of interdependencies (see Table 2). Speakers producing a great amount of motion (i.e., high sum of amplitudes) also tended to display smoother movements (i.e., low expressiveness) and less variation in amplitude height (i.e., low turbulence) and motion direction. Also, there was a relationship between expressiveness and variation in motion direction. Consequently, speakers with a tendency to display fast and jerky movements also tended to change motion direction more often. Although interdependencies between these variables suggest an additional reduction of measures, the filtered motion cues provide insights that cannot be explained solely by common variance (see next paragraphs). Measuring the turbulence index, for instance, reveals that speakers who are less active tend to show more variation in activity, which is different from being less active by just producing smaller amplitudes. As already mentioned it is difficult to assign behavior to independent categories, yet it is clear and also reflected in the results that some behaviors cannot be performed simultaneously. For instance, speakers that predominately display a great deal of vertical movements with the arms will not show much variation in motion direction.

Relations of the extracted motion cues with the Big Five personality dimensions and the variables of career progress are presented as multiple regressions (i.e., with Big Five and measures of career progress as dependent variables; see Table 4) but also as simple bivariate correlations (see Table 3). The latter was done to provide an additional source of information, because the strong interdependencies between measures of motion quality (see also variance inflation factors in Table 4) affect the regression coefficients (i.e., β-weights) of the multiple regressions and this undermines their interpretability and the interpretability of the multiple correlations. In addition, structure coefficients were determined (i.e., bivariate correlations divided by multiple regressions), which provide insight into the underlying structure of the used independent variables (i.e., predictors) by giving an estimate of a single predictor's influence onto the dependent variable without including the remaining predictors (Courville & Thompson, 2001). Although “full light” visual stimuli and a simplified methodology were used, most of the results presented below are in accordance with previous findings based on stick figure animations (Koppensteiner & Grammer, 2010).

The strong association between high overall activity and extraversion, for instance, could be replicated in this study. Such a relationship has even been found for simple animations of a ball moving along the trajectory of a sine wave (Koppensteiner, 2011) and supports findings of extraversion being a trait that is readily attributed to conspicuous behavioral cues (e.g., Kenny et al., 1992). As also shown in our previous stick figure study low activity in the vertical direction (i.e., mostly less sweeping vertical arm movements) was preferably associated with high agreeableness as well as high activity along the horizontal axis (e.g., swaying of upper body) was associated with high emotional stability.

Speakers exhibiting periods of low activity interrupted by periods of high activity (i.e., high turbulence coefficient) along the horizontal and vertical axis tended to be perceived as highly agreeable, while high extraversion was associated with movements showing a low “horizontal” turbulence intensity (i.e., activity with small variation in body moving sideways). Although this is for the most part in a line with previous findings, it also provides additional information, because we did not measure horizontal and vertical turbulence in our stick figure study.

Speakers who produced fast and jerky movements (i.e., high expressiveness) in vertical and horizontal direction were perceived as less emotionally stable than speakers with a tendency to produce slow and smooth movements (i.e., low expressiveness). Similar results had also been provided in our stick figure study. In addition, jerky as opposed to smooth motion patterns even seem to elicit different attributions of emotional stability, when animations are reduced to a simple ball moving along a sine wave (Koppensteiner, 2011).

More complex body movements, in which a great number of changes in motion direction were observed, were preferably associated with high openness, while movements with less variation in motion direction and more “redundancy” were associated with low values on this personality trait.

Analyses of the relations between motion cues and autobiographical data provided less convincing results than correlations between personality ratings and autobiographical data (see Tables 1, 3 and 4). Horizontal turbulence and the sum of vertical amplitudes provided noteworthy findings with career progress, as estimated by years needed to become a member of the “Bundestag”. These measures are related to perceived extraversion, which indicates that politicians communicating extraversion might achieve higher positions at an earlier stage of their career. Such an interpretation, however, should be treated with caution, because the relationship between ratings of extraversion and career progress was less clear. Therefore, it is possible that these motion cues create impressions that cannot be covered by the personality dimensions of the questionnaire used in this study.

## Conclusions

The results obtained have several implications. First, movements of the hands, the head, and the torso of a speaker's upper body provide sufficient information to predict people's impressions to a certain degree. In addition, a small set of landmarks simplifies and reduces workload for behavior encoding. Second, even when cues from other visual communication channels (e.g., appearance cues, facial expressions etc.) are available to observers, the influence of motion cues does not seem to disappear. Therefore, depending on the research question motion behavior may also be analyzed without creating abstract representations such as stick figure or point light animations. Third, it is conceivable predictions can be made on a mathematical basis without using judges for behavior description, thereby building a bridge to automated behavior analyses. With a larger database it could be possible then to create a software based “impression classifier” that is able to classify perceived qualities of a speaker by his or her motion behavior. This, of course, requires further considerations on which motion cues are useful and an extension of the current repertoire of measures. Finally, motion cues seem to play an important or even a predominant role in social judgments of brief displays of behavior. This might be all the more true, when people watch a speaker on a stage or at an election rally, because from a great distance facial expressions and other visual nonverbal cues are difficult to discern. Although results with autobiographical data hint that there is a link, at the current stage of the research it is unclear how personality ratings and the filtered motion cues are related to the actual personality of a politician. This requires a refinement of the methods and additional data on different aspects of a politician's behavior in public.

It also needs further studies with different types of stimuli in order to test whether the presented methodology identifies cues that have signal value or are just mediators for certain gestures (i.e., gestures that produce certain motion patterns, because politicians display them frequently). Also, it remains unclear how motion interacts with other nonverbal and verbal cues (e.g., facial expressions, speech content, static cues etc.). Do motion cues constitute an own level of communication or is there redundant information, which is also conveyed by other communication channels? This and many other questions still need to be answered.

## General discussion

People often judge others by considering brief displays of behavior (e.g., Ambady et al., 2000). This raises questions concerning the specific cues used to form first impressions. Since there is only minimal visual input, it seems clear that such cues should be simple, conspicuous, and displayed repeatedly (see also Thoresen et al., 2012). For this reason the measures applied in this study were designed to track down and decompose “global descriptors” of body motion. For instance, speakers with a tendency to produce sweeping gestures (i.e., high amplitude) or speakers with a tendency to produce gestures with high velocity will display such movements frequently, thereby communicating a “stable” cue on which people base their judgments. This might not only be true for motion, but also for other types of stimuli (e.g., vocal cues), which have a consistent appearance and are easy to perceive.

Related to these considerations is the question of why a particular pattern of motion is ascribed to a particular personality trait. At the current stage of research the answers can only be speculative. People are highly sensitive to nonverbal cues (e.g., Ambady et al., 2000; Stewart et al., 2009) and with a life time of experience in social interactions they have implicitly learned to link different nonverbal cues to different behavioral tendencies. Consequently, the patterns of motion investigated here are associated with cognitive representations of different personality traits.

A slightly different explanation would be that abstract representations of motion patterns or even static patterns share common properties with abstract representations of personality. Extraversion, for instance, might be linked to high amplitude of motion, because on an abstract level there is a common pattern, namely high stimulation. Agreeableness, which was related to high activity interrupted by low activity, might be equated to stimulation with phases of relaxation. Aggressiveness, on the other hand, might be communicated by vertical movements (i.e., arm movements for the most part), because it might be the easiest way to produce sweeping movements with great vigor. For this reason vertical movements might be an unfriendly signal. In contrast to that, horizontal movements (i.e., mostly done with the whole body) are difficult to be performed with great vigor and therefore they might be related to calmness and emotional stability. Such considerations have a either a physiological background, break personality judgments down to simple perceptual processes, or build a bridge to some concepts of embodied or grounded cognition (e.g., Slepian & Ambady, 2012). As already mentioned above, these are crude speculations, which need support from further studies.

Although one of the current study's aims was to present a simple way of behavior encoding, further experimental work is needed to examine motion patterns in greater detail. For instance, interrelations between different measures of motion quality indicate that some motion cues cannot be displayed simultaneously and that they rarely may be displayed in equal shares throughout a sequence of behaviors. With a larger database and additional measures it could be possible then to classify speakers according to their behaviors and produce results that go beyond correlational analyses. A larger database would also be helpful to extend and refine methods of analyses. Although the methodology applied here allows making some predictions, investigations on the level of single landmarks could yield additional insights into the way different body parts interact. This might support speculations made above and reveal, for instance, that some “nonverbal statements” only can be made with vertical arm movements. Additional measures of body motion will also show whether perceptions of conscientiousness that were related to biographical data can be “inferred” from motion also or whether they are affected by other nonverbal information such as appearance cues.

Nonverbal behavior is less accessible to actors than to observers, and as a consequence hard to regulate (DePaulo, 1992). Hence, the motion patterns identified might be a key to the actual personality of a speaker. Since it will be difficult to obtain self-ratings by politicians, their actual personality can only be revealed indirectly by relating public information about leadership qualities or, as done here, biographical data to observer-ratings of personality. The methods applied in this study only give a rough estimate of the politicians' careers and might be extended. Measures of status, for instance, could be refined by assigning different ranks to different public positions, for it is clear that being a minister is a more important leadership position than a being the mayor of a small town.

The results obtained in this study also provide good reason to extend investigations on “thin slices” of behavior to the analysis of body motion and its interactions with other nonverbal and appearance cues. The current study is a tentative step only, and should be followed by work that examines the interplay of different nonverbal communication channels, how body motion is connected to language and verbal content, whether successful and charismatic leaders speak their mind through their bodies, and how all these variables affect the electability of a person.

## Figures and Tables

**Table 1:** Intercorrelations (*r_s_*) between personality traits and relations with biographical information.

Measuring units	Personality trait				
	Openness	Conscient.	Agreebl.	Emot. stab.	Extrav.
Ratings *M* (*SD*)	215.1 (18.8)	238.8 (23.7)	205.9 (38.2)	205.2 (31.2)	221.9 (28.7)
Cronbach's *α*	.62	.72	.87	.73	.80
Openness	1.00				
Conscientiousness	.53⁎⁎	1.00			
Agreeableness	.43⁎⁎	.01	1.00		
Emotional stability	.38⁎	.04	.57⁎⁎	1.00	
Extraversion	.15	.15	− .13	.28	1.00
Entry into parliament	.33⁎	.45⁎⁎	.00	.07	− .24
Memb. and positions	− .31	− .42⁎⁎	− .11	− .06	.14
Leading positions	− .30	− .17	.08	.00	− .02

Note. Agreebl. = agreeableness; Extrav. = extraversion; Conscient. = conscientiousness; Emot. stab. = emotional stability; *α* = internal consistency of questionnaire; Entry into parliament = year of first entry into parliament — year of first entry into a party (*M* = 16.9, *SD* = 9.2); Memb. and positions = all memberships and public positions presented in online biographies/years of age (*M* = .36, *SD* = .14); Leading positions = all current and previous leading positions presented in online biographies/age (*M* = .09, *SD* = .05); *n* = 40.

⁎ *p* < .05.

⁎⁎ *p* < .01.

**Table 2:** Intercorrelations (*r_s_*) between measures of motion quality.

Measuring units	Motion cues						
	Turb. (horz.)	Turb. (vert.)	Amp. (horz.)	Amp. (vert.)	Expr. (horz.)	Expr. (vert.)	Var.in.mo.dir.
Turbulence horizontal	1.00						
Turbulence vertical	.72⁎⁎	1.00					
Amplitudes horizontal	− .56⁎⁎	− .47⁎⁎	1.00				
Amplitudes vertical	− .60⁎⁎	− .51⁎⁎	.70⁎⁎	1.00			
Expressiveness horizontal	.21	.08	− .67⁎⁎	− .10	1.00		
Expressiveness vertical	.03	− .08	− .45⁎⁎	− .34⁎	.49⁎⁎	1.00	
Variation in direction	.27	.20	− .39⁎	− .17	.48⁎⁎	.32⁎	1.00

Note. horz. = horizontal movements; vert. = vertical movements; higher sum of amplitudes = more activity; high turbulence coefficient = more dispersion in amplitudes; high variation in motion direction = motion switches more often from horizontal to vertical motion direction; high expressiveness = higher velocity corrected for overall activity; *n* = 40.

⁎ *p* < .05.

⁎⁎ *p* < .01.

**Table 3:** Correlations (*r_s_*) of motion cues with personality and biographical data.

Measuring units	Motion cues						
	Amp. (horz.)	Amp. (vert.)	Turb. (horz)	Turb. (vert.)	Expr. (horz.)	Expr. (vert.)	Var.in.mo.dir.
Openness	− .10	− .17	.21	.11	.04	.13	.37⁎
Conscientiousness	− .03	− .08	.25	.10	.06	.09	.16
Agreeableness	− .17	− .41⁎⁎	.35⁎	.33⁎	− .17	− .08	.23
Emotional stability	.35⁎	.11	.06	.22	− .48⁎⁎	− .53⁎⁎	− .06
Extraversion	.53⁎⁎	.64⁎⁎	− .37⁎	− .12	− .23	− .22	− .12
Entry into parliament	− .04	− .29	.33⁎	.11	− .16	− .04	.12
Memb. and positions	.02	.23	− .25	.11	.13	− .06	.11
Leading positions	.02	.07	− .02	.09	.18	.10	.20

Note. Entry into parliament = year of first entry into parliament — year of first entry into a party; Memb. and positions = all memberships and public positions presented in online biographies; Leading positions = all current and previous leading positions presented in online biographies; horz. = horizontal movements; vert. = vertical movements; higher sum of amplitudes (Amp.) = more activity; high turbulence coefficient (Turb.) = more dispersion in amplitudes; high variation in motion direction (Var.in.mo.dir) = motion switches more often from horizontal to vertical motion direction; high expressiveness (Expr.) = higher velocity corrected for overall activity; *n* = 40.

⁎ *p* < .05.

⁎⁎ *p* < .01.

**Table 4:** Multiple regressions of motion quality with personality and biographical data.

Criterion	Predictors (β-weights and structure coefficients)							
	*R_mult_*	Amp. (horz.)	Amp. (vert.)	Turb. (horz)	Turb. (vert.)	Expr. (horz.)	Expr. (vert.)	Var.in.mo.dir.
*VIF*		7.10	4.56	2.70	2.34	4.50	1.87	1.40
Openness	.44	.20 (− .22)	− .13 (− .38)	.20 (.47)	− .07 (.25)	− .11 (.10)	.08 (.29)	.41 (.84)
Conscientiousness	.37	.47 (− .07)	− .09 (− .22)	.43 (.67)	− .05 (.28)	.16 (.17)	.15 (.26)	.10 (.44)
Agreeableness	.57	.01 (− .29)	− .38 (− .70)	.09 (.61)	.03 (.57)	− .30 (− .29)	− .16 (− .14)	.33 (.40)
Emotional stability	.66^⁎⁎^	.21 (.53)	− .02 (.17)	− .02 (.09)	.27 (.33)	− .30 (− .72)	− .34 (− .80)	.22 (− .09)
Extraversion	.74^⁎⁎^	.08 (.72)	.79 (.87)	− .14 (− .50)	.46 (− .16)	− .23 (− .31)	.23 (− .29)	.03 (− .16)
Entry parliament	.52	.28 (− .08)	− .37 (− .56)	.48 (.64)	− .32 (.21)	− .15 (− .32)	− .07 (− .01)	.19 (.23)
Memb. a. positions	.57	− .14 (.03)	.23 (.40)	− .70 (− .44)	.62 (.19)	.14 (.23)	− .08 (− .10)	.12 (.19)
Leading positions	.39	.64 (.04)	− .20 (.19)	− .17 (− .04)	.34 (.23)	.50 (.46)	.07 (.25)	.12 (.50)

Note. *R_mult_* = multiple regression coefficient; structure coefficients = *r_xy_* / *R_mult_* (reported in parenthesis); *df1* = 7; *df2* = 32; *VIF* = variance inflation factor; see Table 3 for other abbreviations; *n* = 40. ⁎ *p* < .05. ⁎⁎ *p* < .01.

## Footnotes

1 A brief explanation of this and samples of the used code can be found online by searching for “An Introduction to OpenCV. Displaying and Manipulating Video and Motion Data”.
