# Supplementary figures and images for: Motion cues that make an impression: Predicting perceived personality by minimal motion information
Source: J Exp Soc Psychol. 2013 Nov;49(6):1137–43. doi: 10.1016/j.jesp.2013.08.002 (PMC3819996; doi:10.1016/j.jesp.2013.08.002)

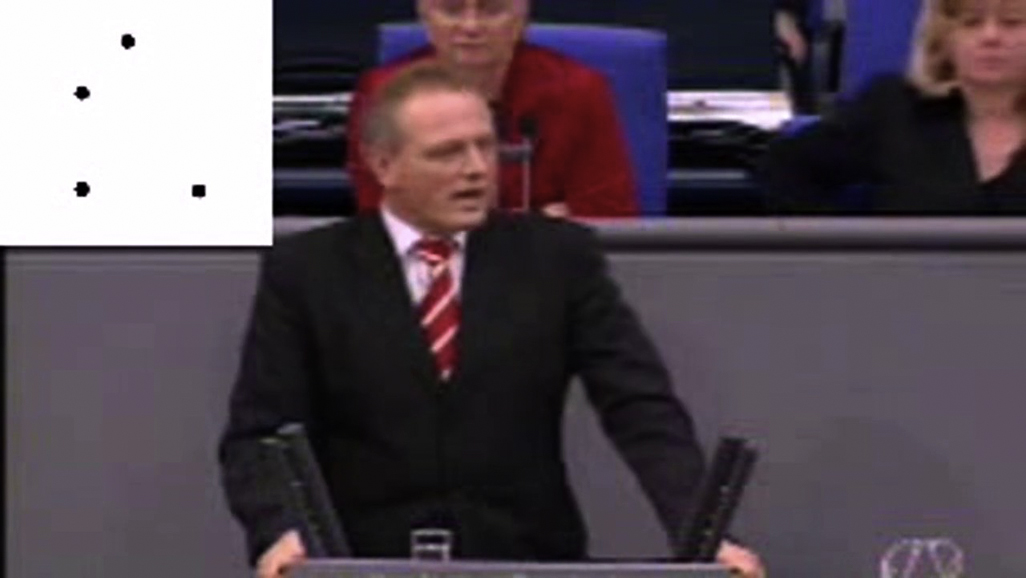

Supplement: Supplementary file 1 — Supplementary video. [file mmc1.jpg]
